# Masked Nocturnal Hypertension and Hidden Organ Risk: A Systematic Review

**DOI:** 10.7759/cureus.111479

**Published:** 2026-06-25

**Authors:** Anas M Berro, Mostafa M Obeid Alla, Mohamed Madani, Rayan E Elkhair, Eithar Musa, Morad Mohammad Ahourani, Asim Ahmed

**Affiliations:** 1 Medicine, Faculty of Medicine, Beirut Arab University, Beirut, LBN; 2 General Medicine, University of Medical Sciences and Technology, Khartoum, SDN; 3 Medicine and Surgery, University of Gezira, Wad Madani, SDN; 4 Medical Education, Hamad General Hospital, Doha, QAT; 5 General Medicine, Alzaiem Alazhari University, Khartoum, SDN; 6 General Practice, Doha Clinic Hospital, Doha, QAT; 7 Internal Medicine, Kharkiv National Medical University, Kharkiv, UKR

**Keywords:** ambulatory blood pressure monitoring, cardiovascular outcomes, isolated nocturnal hypertension, left ventricular hypertrophy, masked nocturnal hypertension

## Abstract

Masked nocturnal hypertension is a hidden blood pressure phenotype in which office blood pressure appears normal or controlled while nighttime ambulatory blood pressure remains elevated. This systematic review evaluated the association between masked nocturnal hypertension or isolated nocturnal hypertension detected by 24-hour ambulatory blood pressure monitoring and cardiovascular or target-organ outcomes in adults with normal or controlled office blood pressure. A systematic search was conducted across PubMed, Web of Science East Mediterranean Region (WOS-EMR), Web of Science, Scopus, and Cochrane Library. Eligible studies included adults with normal or controlled office blood pressure who underwent 24-hour ambulatory blood pressure monitoring and had reported cardiovascular outcomes or target-organ damage. Reviews, editorials, pediatric studies, pregnancy-related studies, studies without nighttime blood pressure data, and studies without relevant outcomes were excluded. Study selection, data extraction, and risk-of-bias assessment were performed using predefined criteria. The included evidence covered population-based cohorts, chronic kidney disease cohorts, diabetes cohorts, hypertension clinic samples, and untreated outpatient populations. Across studies, masked nocturnal hypertension and isolated nocturnal hypertension were generally associated with higher cardiovascular, stroke, and mortality risks, as well as renal, cardiac, vascular, and cerebrovascular target-organ damage, including renal impairment, albuminuria or proteinuria, left ventricular hypertrophy, increased left ventricular mass, arterial stiffness, carotid intima-media thickness, and silent cerebrovascular lesions. However, interpretation should remain cautious because studies differed in population, nocturnal blood pressure thresholds, treatment status, follow-up duration, and outcome definitions. Masked nocturnal hypertension detected by ambulatory blood pressure monitoring appears to be a clinically important phenotype among adults with normal or controlled office blood pressure. The findings support nighttime blood pressure assessment as a useful approach for improving cardiovascular and target-organ risk stratification, especially in high-risk groups such as chronic kidney disease, diabetes, and treated hypertension.

## Introduction and background

Hypertension remains one of the most important modifiable risk factors for cardiovascular disease, stroke, kidney disease, and premature mortality worldwide. Global pooled analyses show that the number of adults aged 30 to 79 years living with hypertension doubled between 1990 and 2019, highlighting the continuing public health burden of elevated blood pressure and the need for accurate detection and risk classification despite advances in diagnosis and treatment [1]. Although hypertension assessment has depended mainly on office or clinic blood pressure, contemporary guidelines increasingly emphasize that office readings alone may not fully represent a patient’s blood pressure burden across the day and night [2-4]. Ambulatory blood pressure monitoring addresses this limitation by providing daytime, nighttime, and 24-hour blood pressure patterns that cannot be captured by clinic readings alone [5]. Accurate blood pressure measurement is not only a technical issue, but also a central step in cardiovascular risk classification and treatment decision-making.

Ambulatory blood pressure monitoring records blood pressure repeatedly over 24 hours during usual daily activities and sleep, allowing identification of circadian blood pressure patterns, nocturnal blood pressure, dipping status, and masked hypertension phenotypes [5]. Masked hypertension is generally defined as normal office blood pressure with elevated out-of-office blood pressure. Masked uncontrolled hypertension describes treated patients whose office blood pressure appears controlled but whose out-of-office blood pressure remains elevated [6]. Nocturnal hypertension refers to elevated blood pressure during sleep, commonly defined by an average nighttime ambulatory blood pressure of at least 120/70 mmHg. Isolated nocturnal hypertension refers to elevated nighttime blood pressure with normal daytime blood pressure [7]. These definitions are clinically important because nighttime blood pressure reflects autonomic regulation, sodium handling, sleep-related physiology, vascular stiffness, and renal pressure control, making nocturnal blood pressure a potentially sensitive marker of hidden cardiovascular and renal risk.

The main clinical problem is that many adults may appear normotensive or well controlled during clinic assessment while still having elevated nighttime blood pressure on ambulatory blood pressure monitoring. This creates a diagnostic blind spot, especially in high-risk groups such as patients with chronic kidney disease, diabetes mellitus, obesity, sleep-related breathing disorders, and treated hypertension. Large ambulatory blood pressure cohorts have shown that nighttime blood pressure has strong prognostic value for mortality and cardiovascular events, and may exceed daytime or office blood pressure for risk prediction [8]. Evidence from hypertensive populations has also shown that nighttime blood pressure and abnormal night-to-day blood pressure patterns are associated with death and cardiovascular events, supporting the importance of nocturnal blood pressure as more than a secondary measurement [9]. Further analyses suggest that nighttime blood pressure remains a meaningful predictor even after accounting for 24-hour blood pressure and dipping pattern, although the degree of added prognostic value may vary between populations and adjustment models [10].

Previous reviews have examined isolated nocturnal hypertension and its relationship with subclinical target-organ damage, including cardiac, renal, and vascular abnormalities [11]. Meta-analytic evidence has shown that masked uncontrolled hypertension is associated with higher cardiovascular event and all-cause mortality risk compared with controlled hypertension, reinforcing the clinical importance of blood pressure phenotypes that are missed by office measurements [12]. However, existing literature has often focused on masked hypertension broadly, combined daytime and nighttime phenotypes, or included populations with uncontrolled office blood pressure. As a result, the specific clinical significance of masked nocturnal hypertension or isolated nocturnal hypertension in adults whose office blood pressure is normal or controlled remains insufficiently synthesized.

In clinical practice, decisions to request ambulatory blood pressure monitoring, intensify treatment, adjust medication timing, or monitor target-organ damage often depend on whether nocturnal blood pressure adds meaningful risk information beyond office blood pressure. Existing studies differ in population, ambulatory blood pressure thresholds, treatment status, follow-up duration, and outcome definitions, which makes it difficult for clinicians to interpret the true burden of masked nocturnal hypertension across cardiovascular and target-organ outcomes. Therefore, a focused synthesis is needed to clarify whether elevated nighttime blood pressure in the presence of normal or controlled office blood pressure identifies a distinct high-risk phenotype.

The objective of this systematic review was to evaluate the association between masked nocturnal hypertension or isolated nocturnal hypertension detected by 24-hour ambulatory blood pressure monitoring and cardiovascular or target-organ outcomes in adults with normal or controlled office blood pressure. The review also aimed to assess associations with major cardiovascular outcomes, mortality, renal outcomes, cardiac target-organ damage, and vascular target-organ markers, and to compare these risks with adults who had normal nighttime blood pressure.

## Review

Methods

Study Design

This systematic review was conducted and reported in line with the Preferred Reporting Items for Systematic Reviews and Meta-Analyses (PRISMA) 2020 statement [13] to evaluate the association between masked nocturnal hypertension or isolated nocturnal hypertension detected by 24-hour ambulatory blood pressure monitoring and cardiovascular or target-organ outcomes in adults with normal or controlled office blood pressure. The review question, eligibility criteria, outcomes, and final scope were based on the predefined proposal, which focused on adults with normal or controlled office blood pressure but elevated nighttime blood pressure on ambulatory blood pressure monitoring compared with adults with normal nighttime blood pressure.

Protocol and registration

The protocol was not prospectively registered in PROSPERO or any other systematic review registry.

PECO Framework

The review question was structured according to the PECO (Population, Exposure, Comparator, Outcomes) framework (Table 1).

**Table 1:** PECO framework of the systematic review. PECO, Population, Exposure, Comparator, Outcomes; ABPM, ambulatory blood pressure monitoring; eGFR, estimated glomerular filtration rate; CKD, chronic kidney disease.

Component	Definition
Population	Adults aged 18 years or older with normal or controlled office or clinic blood pressure.
Exposure	Masked nocturnal hypertension, isolated nocturnal hypertension, masked nighttime hypertension, or nighttime masked uncontrolled hypertension detected by 24-hour ambulatory blood pressure monitoring.
Comparator	Adults with normal office blood pressure and normal nighttime blood pressure, or adults with controlled office blood pressure without nocturnal hypertension.
Outcomes	Cardiovascular events, stroke, myocardial infarction, heart failure, cardiovascular mortality, all-cause mortality, left ventricular hypertrophy, albuminuria or proteinuria, reduced estimated glomerular filtration rate, chronic kidney disease progression, carotid intima-media thickness, arterial stiffness, and pulse wave velocity.

Search Strategy and Data Sources

A systematic literature search was performed in five databases: PubMed, WOS-EMR, Web of Science, Scopus, and Cochrane Library. The final search was conducted on May 21, 2026. The search combined terms related to masked nocturnal hypertension, isolated nocturnal hypertension, nocturnal hypertension, ambulatory blood pressure monitoring, controlled office blood pressure, cardiovascular outcomes, and target-organ damage. The search strategy was adapted according to the syntax of each database (Table 2).

**Table 2:** Database-specific search strategy. WOS-EMR, Web of Science East Mediterranean Region; ABPM, ambulatory blood pressure monitoring; TS, topic search; TITLE-ABS-KEY, title, abstract, and keyword search.

Database	Search string
PubMed	("masked nocturnal hypertension" OR "isolated nocturnal hypertension" OR "nocturnal hypertension" OR "masked nighttime hypertension" OR "nighttime masked uncontrolled hypertension") AND ("ambulatory blood pressure monitoring" OR ABPM OR "24-hour blood pressure") AND ("controlled office blood pressure" OR "normal office blood pressure" OR normotension OR "clinic blood pressure") AND (cardiovascular OR stroke OR mortality OR "target organ damage" OR "left ventricular hypertrophy" OR albuminuria OR proteinuria OR "chronic kidney disease" OR "arterial stiffness" OR "pulse wave velocity" OR "carotid intima-media thickness")
WOS-EMR	("masked nocturnal hypertension" OR "isolated nocturnal hypertension" OR "nocturnal hypertension" OR "masked hypertension") AND ("ambulatory blood pressure monitoring" OR ABPM) AND ("controlled office blood pressure" OR normotension OR "clinic blood pressure") AND (cardiovascular OR stroke OR mortality OR "target organ damage" OR "left ventricular hypertrophy" OR albuminuria OR "arterial stiffness")
Web of Science	TS=("masked nocturnal hypertension" OR "isolated nocturnal hypertension" OR "nocturnal hypertension" OR "masked nighttime hypertension") AND TS=("ambulatory blood pressure monitoring" OR ABPM OR "24-hour blood pressure") AND TS=("controlled office blood pressure" OR "normal office blood pressure" OR normotension OR "clinic blood pressure") AND TS=(cardiovascular OR stroke OR mortality OR "target organ damage" OR "left ventricular hypertrophy" OR albuminuria OR proteinuria OR "arterial stiffness" OR "pulse wave velocity")
Scopus	TITLE-ABS-KEY("masked nocturnal hypertension" OR "isolated nocturnal hypertension" OR "nocturnal hypertension" OR "masked nighttime hypertension") AND TITLE-ABS-KEY("ambulatory blood pressure monitoring" OR ABPM OR "24-hour blood pressure") AND TITLE-ABS-KEY("controlled office blood pressure" OR "normal office blood pressure" OR normotension OR "clinic blood pressure") AND TITLE-ABS-KEY(cardiovascular OR stroke OR mortality OR "target organ damage" OR "left ventricular hypertrophy" OR albuminuria OR proteinuria OR "arterial stiffness" OR "pulse wave velocity")
Cochrane Library	("masked nocturnal hypertension" OR "isolated nocturnal hypertension" OR "nocturnal hypertension" OR "masked hypertension") AND ("ambulatory blood pressure monitoring" OR ABPM) AND ("controlled office blood pressure" OR normotension OR "clinic blood pressure") AND (cardiovascular OR stroke OR mortality OR "target organ damage" OR "left ventricular hypertrophy" OR albuminuria OR "arterial stiffness")

Eligibility Criteria

Studies were included if they enrolled adults aged 18 years or older with normal or controlled office or clinic blood pressure and evaluated masked nocturnal hypertension, isolated nocturnal hypertension, masked nighttime hypertension, or nighttime masked uncontrolled hypertension using 24-hour ambulatory blood pressure monitoring. Studies were eligible if they reported cardiovascular outcomes or target-organ damage outcomes, including cardiovascular events, stroke, myocardial infarction, heart failure, cardiovascular mortality, all-cause mortality, left ventricular hypertrophy, albuminuria, proteinuria, reduced estimated glomerular filtration rate, chronic kidney disease progression, carotid intima-media thickness, arterial stiffness, or pulse wave velocity. Eligible study designs included prospective cohort studies, retrospective cohort studies, registry-based cohort studies, cross-sectional studies reporting target-organ damage, and baseline analyses from cohort studies.

Reviews, systematic reviews, meta-analyses, editorials, letters, protocols, and guidelines were excluded as primary studies. Pediatric studies, pregnancy-related hypertension studies, studies without ambulatory blood pressure monitoring, studies without nighttime blood pressure data, studies reporting daytime hypertension only, studies with uncontrolled office blood pressure without a clearly separable controlled-office subgroup, and studies without cardiovascular or target-organ outcomes were excluded.

Data Extraction

Two reviewers independently extracted the data from the included studies. Extracted variables included first author, publication year, country, study design, sample size, follow-up duration, population characteristics, office and ambulatory blood pressure status, definition of masked nocturnal hypertension or isolated nocturnal hypertension, comparator group, outcomes, effect estimates, adjusted confounders where reported, main findings, and risk-of-bias judgment. Disagreements were resolved through discussion.

Risk-of-Bias Assessment

Risk of bias was assessed according to the study design. Cohort, registry-based, and longitudinal observational studies were assessed using the Newcastle-Ottawa Scale [14]. Cross-sectional studies and observational target-organ-damage studies without follow-up were assessed using the Joanna Briggs Institute critical appraisal checklist for analytical cross-sectional studies [15]. Risk-of-bias judgments considered participant selection, exposure definition, outcome measurement, adjustment for confounders, follow-up adequacy when applicable, and clarity of ambulatory blood pressure monitoring-based nocturnal blood pressure classification.

Data Synthesis

A narrative synthesis was conducted to summarize the included studies by study design, population, ambulatory blood pressure monitoring phenotype, comparator group, and outcome domain. Outcomes were grouped as cardiovascular events, stroke, mortality, renal outcomes, cardiac target-organ damage, vascular damage, and cerebrovascular damage. Clinical and methodological heterogeneity reflected differences in population type, treatment status, nocturnal blood pressure definition, ambulatory blood pressure monitoring thresholds, follow-up duration, and outcome measurement.

Quantitative pooling was not performed because the included studies differed substantially in exposure definitions, population characteristics, comparator groups, outcome domains, and reported effect measures. Therefore, findings were synthesized narratively, with emphasis on consistency of direction, outcome domain, population type, and methodological quality. This approach was consistent with Cochrane guidance that synthesis should account for heterogeneity and that statistical pooling may be inappropriate when studies are not sufficiently comparable [16].

Results

Study Selection

The search identified 494 records. Before screening, 233 records were removed as duplicates or clearly irrelevant records. A total of 261 records remained for title and abstract screening. Of these, 197 records were excluded because they were not directly related to ambulatory blood pressure monitoring, masked nocturnal hypertension, isolated nocturnal hypertension, controlled or normal office blood pressure, cardiovascular outcomes, or target-organ damage. Therefore, 64 full-text articles were assessed for eligibility.

**Figure 1:**
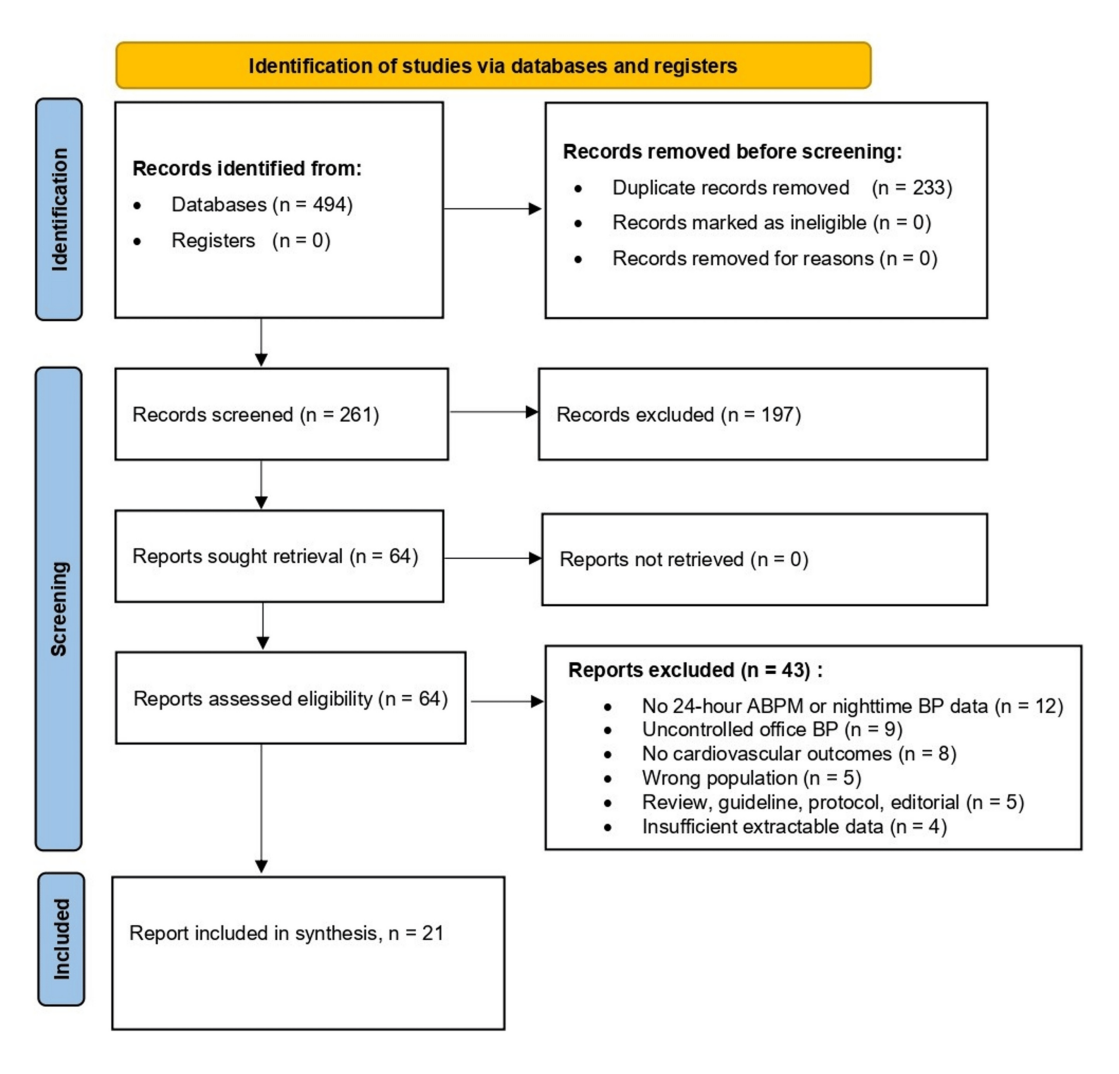
PRISMA flow chart of study selection (n = 494 records). PRISMA, Preferred Reporting Items for Systematic Reviews and Meta-Analyses; ABPM, ambulatory blood pressure monitoring; MNH, masked nocturnal hypertension; INH, isolated nocturnal hypertension.

After full-text assessment, 43 articles were excluded. The main reasons for exclusion were absence of 24-hour ambulatory blood pressure monitoring or nighttime blood pressure data (n = 12), uncontrolled office blood pressure without a clearly separable controlled-office subgroup (n = 9), absence of cardiovascular or target-organ damage outcomes (n = 8), wrong population such as pediatric, pregnancy-related, or non-relevant clinical groups (n = 5), review articles, guidelines, protocols, editorials, or letters (n = 5), and insufficient extractable data for the review question (n = 4). Finally, 21 studies were included in the qualitative synthesis (Figure 1).

Study Characteristics

The final synthesis included 21 studies published between 2002 and 2025. The studies represented different clinical and population settings, including type 2 diabetes mellitus cohorts, chronic kidney disease cohorts, hypertension clinic samples, untreated outpatient populations, registry-based populations, and large population-based cohorts. The geographical distribution was broad and included studies from Sweden, China, Romania, Italy, Japan, South Korea, Vietnam, Spain, and the United States, in addition to multinational cohorts.

The included studies used observational designs. Prospective cohort and registry-based studies mainly assessed cardiovascular events, stroke, renal events, all-cause mortality, and cardiovascular mortality. Cross-sectional and retrospective observational studies mainly evaluated subclinical target-organ damage, including left ventricular hypertrophy, left ventricular mass index, arterial stiffness, pulse wave velocity, carotid intima-media thickness, albuminuria, proteinuria, reduced estimated glomerular filtration rate, and silent cerebrovascular lesions.

Most studies defined nocturnal hypertension using a nighttime ambulatory blood pressure threshold close to ≥120/70 mmHg, although some studies used slightly different nighttime thresholds or evaluated nocturnal systolic blood pressure as a continuous or grouped exposure. The main exposure phenotypes were masked nocturnal hypertension, isolated nocturnal hypertension, isolated nighttime masked uncontrolled hypertension, masked nighttime hypertension, nocturnal masked hypertension, and nocturnal hypertension within broader masked hypertension patterns. Full descriptive characteristics of the included studies are provided in Table 3.

**Table 3:** Characteristics of included studies evaluating masked nocturnal hypertension and related nocturnal blood pressure phenotypes (n = 21 studies). ABPM, ambulatory blood pressure monitoring; BP, blood pressure; CKD, chronic kidney disease; CIMT, carotid intima-media thickness; CV, cardiovascular; dMHT, daytime masked hypertension; dnMHT, day-night masked hypertension; eGFR, estimated glomerular filtration rate; HR, hazard ratio; HTN, hypertension; INH, isolated nocturnal hypertension; KoGES, Korean Genome and Epidemiology Study; LVH, left ventricular hypertrophy; LVMI, left ventricular mass index; MH, masked hypertension; MNH, masked nocturnal hypertension; MNHT, masked nocturnal hypertension; MUCH, masked uncontrolled hypertension; NH, nocturnal hypertension; nMHT, nocturnal masked hypertension; OR, odds ratio; PWV, pulse wave velocity; RWT, relative wall thickness; SBP, systolic blood pressure; T2DM, type 2 diabetes mellitus; TOD, target-organ damage; UACR, urinary albumin-to-creatinine ratio; WMH, white matter hyperintensity.

Study ID	Country/setting	Design	N	Follow-up	Population/BP status	Exposure/MNH definition	Comparator	Outcomes	Main finding/effect estimate	Notes
Wijkman M et al., 2009 [17]	Sweden	Cross-sectional	414	None	Adults with T2DM; clinic and ABPM measured	MNHT: clinic BP <130/80 mmHg with nighttime BP ≥120/70 mmHg	Clinical and nocturnal normotension	PWV, central BP	MNHT found in 7.2%; MNHT had higher aortic PWV and central BP	Strong fit for arterial stiffness
Fu X et al., 2022 [18]	China	Retrospective cohort	675	Median 39 months	Nondialysis CKD with hypertension	MUCH divided into isolated nighttime MUCH and day-night MUCH using office and ABPM	Controlled hypertension	LVH, composite kidney outcome	MUCH associated with LVH, OR 2.94; isolated nighttime MUCH associated with kidney outcome, HR 4.27	Strong CKD outcome study
Călin P et al., 2022 [19]	Romania	Prospective observational	163	Not reported in abstract	Treated hypertensive T2DM with apparently controlled office BP	Isolated nocturnal MUCH based on elevated mean nighttime ABPM	Dippers/extreme dippers without MUCH	Prevalence, BP pattern, associated factors	49.7% had isolated nocturnal MUCH; associated factors included older age, longer DM, longer HTN, obesity, CV comorbidity	Descriptive; weaker for hard outcomes
Fan HQ et al., 2010 [20]	10 populations	Prospective cohort	8711	Not stated in abstract	Randomly recruited adults; untreated subgroup analyzed	INH: daytime BP <135/85 and nighttime BP ≥120/70 mmHg	Ambulatory normotension / isolated daytime HTN	Hard cardiovascular endpoints	INH predicted hard cardiovascular endpoints independently of conventional risk factors	Strong prognostic evidence
Wang C et al., 2016 [21]	China	Cohort	588	Not stated in abstract	Nondialysis CKD	INH among nocturnal hypertension group	Normotension / nocturnal normotension	Total mortality, CV mortality, renal events, CV events	INH associated with renal events, HR 2.78, and CV events, HR 6.82	Strong CKD prognosis study
Presta V et al., 2018 [22]	Italy	Retrospective cohort/clinic database	2628 untreated adults; 153 MH	Not clearly stated	Untreated adults with masked hypertension	MH with nocturnal dipping, non-dipping, reverse dipping patterns	Dippers and non-dippers	Stroke and CV outcomes	Reverse dipping associated with higher stroke risk, OR 18.660	Useful for nocturnal pattern risk
Li X et al., 2021 [23]	China	Cross-sectional	2386	None	Nondialysis CKD	Isolated nocturnal hypertension and combined morning/nocturnal hypertension by ABPM	No morning/nocturnal HTN	LVH, CIMT, eGFR, albuminuria	Isolated NH and combined MH/NH associated with cardiovascular and renal damage; combined group had higher LVH, CIMT, low eGFR, albuminuria	Strong TOD study
Brguljan-Hitij J et al., 2014 [24]	11 populations	Prospective cohort	7826	Mean 11.3 years	Untreated adults across JNC office BP classes	Masked hypertension by ABPM; mainly daytime threshold in abstract	True normotension	Mortality, CV, cardiac, cerebrovascular events	Masked hypertension increased stroke risk in normotension and prehypertension	Supportive ABPM risk study; not purely nocturnal
de la Sierra A et al., 2025 [25]	Spain	Registry cohort	4999 masked HTN plus 10006 normal BP	Median 9.7 years	Patients with normal office BP and elevated ambulatory BP	Isolated nighttime masked HTN, isolated daytime masked HTN, day-night masked HTN	Normal office and 24-hour BP	All-cause death, CV death	Isolated nighttime masked HTN increased death risk, HR 1.39; day-night masked HTN HR 1.22	Strong mortality evidence
Carollo C et al., 2025 [26]	Italy	Retrospective observational	1340	None	Hypertension center cohort	INH by ABPM	Non-INH / normal BP subgroup	eGFR, AER, CKD	INH prevalence 11%; AER and eGFR independently associated with INH	Renal target-organ evidence
Ogedegbe G et al., 2013 [27]	USA	Population-based cross-sectional	425	None	African American adults from Jackson Heart Study	INH by 24-hour ABPM	Normotension	LV mass, LVH, proteinuria	INH associated with higher LV mass in age/sex models; multivariable adjustment weakened significance	Useful population-based TOD evidence
Hoshide S et al., 2007 [28]	Japan	Cross-sectional	165	None	Community-dwelling hypertensive subjects with controlled home BP	Masked nocturnal HTN: home BP <135/85 and nocturnal ABPM ≥120/75	Home and nocturnal normotension	IMT, RWT	Masked nocturnal HTN had greater IMT and RWT	Borderline due home BP comparator
Li Y et al., 2007 [29]	China	Population cross-sectional	677	None	JingNing population study	INH: nighttime BP ≥120/70 with daytime BP <135/85	Ambulatory normotension	Arterial stiffness indices	INH prevalence 10.9%; associated with increased arterial stiffness	Foundational INH clinical entity study
Ohkubo T et al., 2002 [30]	Japan	Prospective cohort	1542	Mean 9.2 years	General population aged ≥40 years	Nocturnal BP decline rather than MNH/INH	Different nocturnal decline levels	CV mortality	Each 5% lower nocturnal BP decline linked to about 20% higher CV mortality	Supportive nocturnal BP evidence, not core MNH
Booth JN 3rd et al., 2016 [31]	USA	Prospective cohort	738	Median 8.2 years for CVD events	Black adults with clinic BP <140/90 and ABPM	Masked nighttime HTN: nighttime BP ≥120/70	No masked hypertension	CVD events, mortality	Masked nighttime HTN associated with CVD events, HR 2.35	Strong CVD outcome study
Drawz PE et al., 2016 [32]	USA	Cross-sectional	1492	None	CKD patients from CRIC	Masked HTN and elevated nighttime BP	Controlled BP	eGFR, proteinuria, LVMI, PWV	Masked HTN associated with lower eGFR, proteinuria, LVMI, and PWV; lower eGFR seen with elevated nighttime BP	Strong CKD/TOD evidence
Zhang DY et al., 2020 [33]	China	Cross-sectional	1808	None	Untreated outpatients referred for ABPM	Daytime masked hypertension (dMHT), nocturnal masked hypertension (nMHT), and day-night masked hypertension (dnMHT) using office and ABPM thresholds	Normotension	cfPWV, cIMT, LVMI, E/E’, eGFR, UACR	nMHT had thicker cIMT and increased UACR; dnMHT had broader TOD burden	Strong subtype evidence
Kim SH et al., 2022 [34]	Korea	Population-based observational	1734	Not stated in abstract	KoGES participants	INH by ABPM	Normotension	PWV, LVMI, diastolic function, WMH	INH associated with higher PWV, central SBP, LVMI, worse diastolic function, and WMH presence	Strong heart/brain TOD evidence
Li J et al., 2019 [35]	China	Observational	721	None	Untreated nocturnal MH patients	Nocturnal systolic, diastolic, or systolic/diastolic MH	Different nocturnal MH patterns	Echocardiographic cardiac damage	Nocturnal SBP independently associated with left atrial dimension, septal thickness, and LV mass	Strong cardiac damage evidence
Zhang J et al., 2021 [36]	China	Cross-sectional	1166	None	Hospitalized nondialysis CKD; normotensive and hypertensive groups	Nocturnal SBP level/tertiles by ABPM	Lower nocturnal SBP tertiles	LVMI, eGFR, proteinuria	Nocturnal SBP independently associated with TOD in normotensive CKD; highest tertile ≥114 mmHg associated with TOD	Supportive nocturnal SBP evidence
Do TM et al., 2025 [37]	Vietnam	Retrospective study	178	None	CKD with controlled office BP	MUCH detected by ABPM; all MUCH had elevated nighttime BP	Controlled hypertension	LVH	MUCH prevalence 48.9%; LVH higher in MUCH; MUCH independently associated with LVH, OR 1.97	Strong direct CKD/LVH evidence

Key Findings and Synthesis

Across the included studies, nocturnal blood pressure abnormalities were frequently detected among adults with normal, controlled, or apparently controlled office blood pressure. In type 2 diabetes mellitus, Wijkman M et al., 2009, reported masked nocturnal hypertension among patients with clinic normotension, while Călin P et al., 2022, found a high prevalence of isolated nocturnal masked uncontrolled hypertension among treated patients with type 2 diabetes and apparently controlled office blood pressure [17,19]. In chronic kidney disease, Fu X et al., 2022; Wang C et al., 2016; Li X et al., 2021; and Do TM et al., 2025 showed that nighttime hypertension or nighttime masked uncontrolled hypertension was common despite controlled or apparently controlled office blood pressure [18,21,23,37].

Cardiovascular outcome studies generally showed a higher risk among patients with nocturnal hypertension phenotypes. Fan HQ et al., 2010, found that isolated nocturnal hypertension predicted hard cardiovascular endpoints in a large multinational cohort [20]. Wang C et al., 2016, reported increased renal and cardiovascular event risk among nondialysis chronic kidney disease patients with isolated nocturnal hypertension [21]. Presta V et al., 2018, linked reverse dipping among masked hypertension patients with higher stroke risk [22]. Brguljan-Hitij J et al., 2014, showed that ambulatory blood pressure monitoring improved risk stratification in conventionally normotensive and prehypertensive individuals, particularly through the identification of masked hypertension [24]. de la Sierra A et al., 2025, showed that isolated nighttime masked hypertension was associated with increased all-cause and cardiovascular mortality, while isolated daytime masked hypertension was not associated with increased death risk [25]. Booth JN 3rd et al., 2016, also found that masked nighttime hypertension was associated with incident cardiovascular disease events [31].

Target-organ damage was consistently reported across cardiac, renal, vascular, and cerebrovascular domains. Cardiac findings included higher left ventricular mass, left ventricular hypertrophy, increased relative wall thickness, and altered left ventricular mechanics or diastolic function in studies by Fu X et al., 2022; Ogedegbe G et al., 2013; Hoshide S et al., 2007; Kim SH et al., 2022; Li J et al., 2019; and Do TM et al., 2025 [18,27,28,34,35,37]. Renal findings included lower estimated glomerular filtration rate, higher albuminuria or proteinuria, and worse renal outcomes in studies by Fu X et al., 2022; Wang C et al.; 2016, Li X et al., 2021; Carollo C et al., 2025; Drawz PE et al., 2016; Zhang DY et al., 2020; and Zhang J et al., 2021 [18,21,23,26,32,33,36].

Vascular and cerebrovascular damage were also reported. Wijkman M et al., 2009, and Li Y et al., 2007, found associations between nocturnal hypertension phenotypes and arterial stiffness [17,29]. Zhang DY et al., 2020, and Kim SH et al., 2022, reported associations with carotid intima-media thickness, pulse wave velocity, left ventricular mass index, and silent cerebrovascular lesions [33,34]. Overall, the findings suggest that nocturnal blood pressure elevation detected by ambulatory blood pressure monitoring may identify a clinically relevant risk phenotype that is not captured by office blood pressure alone. The outcome-level synthesis is summarized in Table 4.

**Table 4:** Summary of key findings across included studies (n = 21 studies). MNH, masked nocturnal hypertension; INH, isolated nocturnal hypertension; BP, blood pressure; ABPM, ambulatory blood pressure monitoring; CKD, chronic kidney disease; LVH, left ventricular hypertrophy; LV, left ventricular; eGFR, estimated glomerular filtration rate.

Outcome domain	Main studies	Summary of findings
Prevalence and detection of MNH or INH	Wijkman M et al., 2009 [17]; Călin P et al., 2022 [19]; Wang C et al., 2016 [21]; Li Y et al., 2007 [29]; Kim SH et al., 2022 [34]; Do TM et al., 2025 [37]	Nocturnal hypertension phenotypes were common in adults with normal, controlled, or apparently controlled office BP, especially in diabetes and CKD populations.
Cardiovascular events and stroke	Fan HQ et al., 2010 [20]; Wang C et al., 2016 [21]; Presta V et al., 2018 [22]; Brguljan-Hitij J et al., 2014 [24]; Booth JN 3rd et al., 2016 [31]	Isolated nocturnal hypertension, masked nighttime hypertension, and adverse nocturnal BP patterns were associated with higher cardiovascular event or stroke risk.
Mortality	de la Sierra A et al., 2025 [25]; Ohkubo T et al., 2002 [30]	Nocturnal BP abnormalities were associated with cardiovascular mortality or all-cause mortality. The strongest subtype evidence came from isolated nighttime masked hypertension.
Cardiac target-organ damage	Fu X et al., 2022 [18]; Ogedegbe G et al., 2013 [27]; Hoshide S et al., 2007 [28]; Kim SH et al., 2022 [34]; Li J et al., 2019 [35]; Do TM et al., 2025 [37]	Nocturnal hypertension phenotypes were linked with LVH, higher LV mass, increased relative wall thickness, and adverse echocardiographic parameters.
Renal target-organ damage	Fu X et al., 2022 [18]; Wang C et al., 2016 [21]; Li X et al., 2021 [23]; Carollo C et al., 2025 [26]; Drawz PE et al., 2016 [32]; Zhang DY et al., 2020 [33]; Zhang J et al., 2021 [36]	Nocturnal BP elevation was associated with albuminuria, proteinuria, reduced eGFR, CKD progression, and renal events.
Arterial stiffness and vascular damage	Wijkman M et al., 2009 [17]; Li X et al., 2021 [23]; Li Y et al., 2007 [29]; Drawz PE et al., 2016 [32]; Zhang DY et al., 2020 [33]; Kim SH et al., 2022 [34]	Studies reported higher pulse wave velocity, arterial stiffness indices, carotid intima-media thickness, and central systolic BP.
Cerebrovascular damage	Presta V et al., 2018 [22]; Brguljan-Hitij J et al., 2014 [24]; Kim SH et al., 2022 [34]	Evidence suggested increased stroke risk or higher likelihood of silent cerebrovascular lesions among patients with nocturnal BP abnormalities.

Risk of Bias

Risk of bias was assessed according to the study design. Cohort and registry-based studies were assessed using the Newcastle-Ottawa Scale [14], while cross-sectional and observational target-organ-damage studies were assessed using the Joanna Briggs Institute Critical Appraisal Checklist for Analytical Cross-Sectional Studies [15]. Overall, the risk of bias ranged from low to moderate. The strongest studies were generally large cohort, registry-based, or population-based studies with clear ambulatory blood pressure monitoring definitions, adjusted analyses, and clinically important outcomes. Studies with higher risk concerns were mainly small single-center studies, clinic-based samples, cross-sectional analyses, or studies where nocturnal hypertension was not the only exposure phenotype.

The detailed risk-of-bias assessment of the included studies is shown in Table 5.

**Table 5:** Risk-of-bias assessment of included studies (n = 21 studies). ABPM, ambulatory blood pressure monitoring; BP, blood pressure; CKD, chronic kidney disease; CVD, cardiovascular disease; INH, isolated nocturnal hypertension; JBI, Joanna Briggs Institute; LVH, left ventricular hypertrophy; MNH, masked nocturnal hypertension; SBP, systolic blood pressure; TOD, target-organ damage.

Study ID	Design category	Appraisal tool	Overall risk of bias	Main considerations
Wijkman M et al., 2009 [17]	Cross-sectional	JBI cross-sectional checklist	Moderate	Clear ABPM exposure and vascular outcomes, but single clinical population and cross-sectional design.
Fu X et al., 2022 [18]	Retrospective cohort	Newcastle-Ottawa Scale	Low to moderate	Clear CKD cohort, adjusted analyses, and outcome follow-up, but retrospective design may introduce residual confounding.
Călin P et al., 2022 [19]	Prospective observational	Newcastle-Ottawa Scale	Moderate	Clear ABPM assessment, but mainly descriptive and focused on prevalence and BP patterns rather than hard outcomes.
Fan HQ et al., 2010 [20]	Prospective cohort	Newcastle-Ottawa Scale	Low	Large multinational cohort, clear ABPM phenotype, and hard cardiovascular endpoints.
Wang C et al., 2016 [21]	Cohort	Newcastle-Ottawa Scale	Low to moderate	Relevant CKD cohort and outcome follow-up, but single-country setting and CKD-specific population.
Presta V et al., 2018 [22]	Retrospective cohort/database study	Newcastle-Ottawa Scale	Moderate	Useful outcome data, but the reverse-dipping subgroup was small.
Li X et al., 2021 [23]	Cross-sectional	JBI cross-sectional checklist	Moderate	Large CKD sample and multiple TOD measures, but no temporal inference.
Brguljan-Hitij J et al., 2014 [24]	Prospective cohort	Newcastle-Ottawa Scale	Low	Large multinational sample and long follow-up, but exposure was broader masked hypertension rather than strictly nocturnal.
de la Sierra A et al., 2025 [25]	Registry cohort	Newcastle-Ottawa Scale	Low	Large registry, long follow-up, direct masked hypertension subtype analysis, and mortality outcomes.
Carollo C et al., 2025 [26]	Retrospective observational	JBI cross-sectional checklist	Moderate	Direct renal markers and ABPM, but retrospective and clinic-based.
Ogedegbe G et al., 2013 [27]	Population-based cross-sectional	JBI cross-sectional checklist	Moderate	Population-based sample and TOD measures, but associations weakened after multivariable adjustment.
Hoshide S et al., 2007 [28]	Cross-sectional	JBI cross-sectional checklist	Moderate to high	Small sample and comparator based on home BP rather than purely office BP.
Li Y et al., 2007 [29]	Population-based cross-sectional	JBI cross-sectional checklist	Moderate	Foundational population evidence, but cross-sectional design and intermediate vascular outcomes only.
Ohkubo T et al., 2002 [30]	Prospective cohort	Newcastle-Ottawa Scale	Moderate	Strong prognostic cohort, but evaluated nocturnal BP decline rather than strict MNH or INH.
Booth JN 3rd et al., 2016 [31]	Prospective cohort	Newcastle-Ottawa Scale	Low to moderate	Clear masked nighttime hypertension definition and CVD events, but restricted to Black adults in one cohort.
Drawz PE et al., 2016 [32]	Cross-sectional	JBI cross-sectional checklist	Moderate	Large CKD cohort and multiple TOD outcomes, but cross-sectional and CKD-specific.
Zhang DY et al., 2020 [33]	Cross-sectional	JBI cross-sectional checklist	Moderate	Clear masked hypertension subtypes and multiple TOD measures, but single-center outpatient sample.
Kim SH et al., 2022 [34]	Population-based observational	JBI cross-sectional checklist	Low to moderate	Population-based data and multiple organ outcomes, but mainly observational without hard event follow-up in the abstract.
Li J et al., 2019 [35]	Observational	JBI cross-sectional checklist	Moderate	Detailed echocardiographic outcomes, but restricted to untreated nocturnal masked hypertension patients.
Zhang J et al., 2021 [36]	Cross-sectional	JBI cross-sectional checklist	Moderate	Relevant CKD nocturnal SBP and TOD data, but exposure was nocturnal SBP level rather than strict MNH.
Do TM et al., 2025 [37]	Retrospective observational	JBI cross-sectional checklist	Moderate	Direct CKD, controlled office BP, ABPM, and LVH assessment, but single-center and retrospective.

Discussion

Principal Findings

This systematic review synthesized evidence from 21 studies evaluating masked nocturnal hypertension, isolated nocturnal hypertension, nighttime masked uncontrolled hypertension, and related nocturnal blood pressure phenotypes detected by ambulatory blood pressure monitoring in adults with normal, controlled, or apparently controlled office blood pressure [17-37]. Overall, the evidence suggests that nocturnal blood pressure elevation is not a benign hidden finding. Across different clinical populations, particularly patients with type 2 diabetes mellitus, chronic kidney disease, and population-based cohorts, nocturnal hypertension phenotypes were associated with cardiovascular events, cardiovascular mortality, all-cause mortality, renal events, left ventricular hypertrophy, arterial stiffness, carotid intima-media thickness, albuminuria, reduced kidney function, and silent cerebrovascular lesions. The central finding is that office blood pressure control did not reliably exclude clinically important nocturnal hypertension or related organ risk.

Wijkman M et al., 2009, showed that patients with type 2 diabetes and normal clinic blood pressure could still have masked nocturnal hypertension, with higher aortic pulse wave velocity and central blood pressure than patients with both clinic and nocturnal normotension [17]. Fu X et al., 2022, similarly showed that nighttime masked uncontrolled hypertension was common among nondialysis chronic kidney disease patients with controlled office blood pressure and was associated with left ventricular hypertrophy and worse kidney outcomes [18]. Călin P et al., 2022, extended this observation to treated patients with type 2 diabetes and apparently controlled office blood pressure, showing that nearly half had isolated nocturnal masked uncontrolled hypertension despite antihypertensive treatment [19]. Together, these findings show that office blood pressure control may hide clinically relevant nocturnal cardiovascular and renal risk.

Cardiovascular Outcomes and Mortality

The strongest evidence for hard cardiovascular outcomes came from large prospective and registry-based studies. Fan HQ et al., 2010, provided important population-level evidence by showing that isolated nocturnal hypertension predicted hard cardiovascular endpoints in 8711 individuals from 10 populations [20]. Wang C et al., 2016, further strengthened this conclusion in nondialysis chronic kidney disease, where isolated nocturnal hypertension was associated with both renal events and cardiovascular events [21]. These findings support isolated nocturnal hypertension as a prognostic phenotype rather than only a measurement abnormality.

Presta V et al., 2018, added a complementary perspective by showing that reverse dipping among patients with masked hypertension was associated with higher stroke risk [22]. This suggests that the nocturnal pattern itself may add prognostic information beyond the general masked hypertension category. However, this finding should be interpreted cautiously because the reverse-dipping subgroup was small and the confidence interval around the stroke estimate was wide. Therefore, the finding is clinically relevant but should not be overinterpreted.

The registry evidence from de la Sierra A et al., 2025, is particularly important because it compared masked hypertension subtypes [25]. Isolated nighttime masked hypertension was associated with increased all-cause and cardiovascular mortality, whereas isolated daytime masked hypertension was not clearly associated with increased mortality. Booth JN 3rd et al., 2016, also showed that masked nighttime hypertension was associated with incident cardiovascular disease events in Black adults with clinic blood pressure below 140/90 mmHg [31]. Together, these studies suggest that nighttime blood pressure elevation may be a key risk component within the broader masked hypertension phenotype.

Ohkubo T et al., 2002, did not directly classify masked nocturnal hypertension, but it provides supportive physiological evidence by showing that reduced nocturnal blood pressure decline was associated with cardiovascular mortality [30]. Therefore, this study is best interpreted as supportive nocturnal blood pressure evidence rather than direct masked nocturnal hypertension evidence.

Target-Organ Damage

The included studies also showed consistent associations between nocturnal hypertension phenotypes and subclinical target-organ damage. Cardiac damage was one of the most frequently reported domains. Fu X et al., 2022, found that masked uncontrolled hypertension in chronic kidney disease was associated with left ventricular hypertrophy, while Do TM et al., 2025, found that masked uncontrolled hypertension was independently associated with left ventricular hypertrophy among CKD patients with controlled office blood pressure [18,37]. These studies are closely aligned because both involved CKD, controlled or apparently controlled office blood pressure, ambulatory blood pressure monitoring, and cardiac structural damage.

Other studies supported the same cardiac signal in broader populations. Ogedegbe G et al., 2013, found that isolated nocturnal hypertension was associated with greater left ventricular mass in age-sex adjusted analyses among African American participants, although this association weakened after multivariable adjustment [27]. Kim SH et al., 2022, showed that isolated nocturnal hypertension was associated with higher left ventricular mass index and worse diastolic function in the Korean Genome and Epidemiology Study (KoGES) cohort [34]. Li J et al., 2019, showed that nocturnal systolic blood pressure was independently associated with left atrial dimension, interventricular septum thickness, and left ventricular mass among untreated patients with nocturnal masked hypertension [35]. These findings suggest that persistent nighttime systolic load may contribute to cardiac remodeling, while still requiring cautious interpretation because the evidence is observational.

Renal target-organ damage was also strongly represented. Fu X et al., 2022, and Wang C et al., 2016, linked nocturnal hypertension phenotypes to adverse renal outcomes in nondialysis CKD [18,21]. Li X et al., 2021, showed that nocturnal hypertension in CKD was associated with markers of both renal and cardiovascular damage, including low estimated glomerular filtration rate, albuminuria, left ventricular hypertrophy, and abnormal carotid intima-media thickness [23]. Carollo C et al., 2025, showed that albumin excretion rate and estimated glomerular filtration rate were independently associated with isolated nocturnal hypertension [26]. Drawz PE et al., 2016, also linked masked hypertension in CKD with lower estimated glomerular filtration rate, proteinuria, increased left ventricular mass index, and higher pulse wave velocity [32]. Overall, the renal evidence suggests that nocturnal blood pressure elevation may be closely linked to glomerular and vascular injury in CKD.

Vascular and cerebrovascular findings followed the same direction. Wijkman M et al., 2009, reported higher aortic pulse wave velocity in patients with masked nocturnal hypertension and type 2 diabetes [17]. Li Y et al., 2007, showed that isolated nocturnal hypertension in a Chinese population was associated with higher arterial stiffness indices [29]. Zhang DY et al., 2020, found that nocturnal masked hypertension was associated with thicker carotid intima-media thickness and increased urinary albumin-to-creatinine ratio, while Kim SH et al., 2022, linked isolated nocturnal hypertension with increased pulse wave velocity and silent cerebrovascular lesions [33,34]. These findings indicate that nocturnal hypertension is associated with a wider vascular injury phenotype rather than a single organ outcome.

Comparison With True Normotension and Controlled Nocturnal Blood Pressure

A repeated pattern across the evidence is that masked nocturnal hypertension and isolated nocturnal hypertension were worse than true normotension, but often not as severe as sustained or combined day-night hypertension. This gradient was clear in Kim SH et al., 2022, where isolated nocturnal hypertension showed worse arterial stiffness, central systolic blood pressure, left ventricular mass index, and diastolic function compared with normotension, while overt diurnal hypertension generally represented a more advanced phenotype [34]. This stepwise pattern supports the biological plausibility of increasing risk as an ambulatory blood pressure abnormality becomes more extensive.

In CKD, this risk gradient appeared sharper. Wang C et al., 2016, showed that isolated nocturnal hypertension carried a higher renal and cardiovascular event risk than normotension [21]. Fu X et al., 2022, showed that nighttime masked uncontrolled hypertension was associated with worse cardiac and kidney outcomes compared with controlled hypertension [18]. Li X et al., 2021, found that isolated nocturnal hypertension was already associated with target-organ damage, while combined morning and nocturnal hypertension showed broader injury across cardiac, vascular, and renal measures [23]. This suggests that isolated nocturnal hypertension may represent an early but clinically meaningful stage on the pathway toward more generalized ambulatory hypertension.

Hoshide S et al., 2007, compared patients with controlled self-measured home blood pressure but elevated nocturnal ambulatory blood pressure against those with controlled home and nocturnal blood pressure [28]. The masked nocturnal hypertension group had greater carotid intima-media thickness and relative wall thickness. Although this study is less directly aligned with the current review because the comparator relied on home rather than office blood pressure, it still supports the added value of nocturnal ambulatory monitoring in identifying hidden organ stress.

Treated Versus Untreated Populations

The evidence suggests that nocturnal hypertension remains clinically relevant in both treated and untreated adults. Untreated population evidence from Fan HQ et al., 2010, and Brguljan-Hitij J et al., 2014, supports the prognostic value of ambulatory hypertension phenotypes before formal treatment effects are introduced [20,24]. In these settings, nocturnal or masked ambulatory hypertension may reveal vascular risk that conventional office categories fail to detect.

In treated patients, Călin P et al., 2022, showed that patients with type 2 diabetes could have controlled office blood pressure while still demonstrating isolated nocturnal masked uncontrolled hypertension [19]. Fu X et al., 2022, and Do TM et al., 2025, showed similar concerns in CKD, where controlled office blood pressure did not exclude nighttime masked uncontrolled hypertension or associated target-organ damage [18,37]. These findings suggest that antihypertensive treatment may reduce clinic blood pressure without fully controlling nighttime blood pressure, especially in high-risk groups such as those with diabetes and CKD.

However, direct treated-versus-untreated comparisons were limited. Most studies either focused on one treatment group or adjusted for treatment status rather than testing whether nocturnal hypertension has different prognostic strength in treated and untreated patients. Therefore, treatment status remains an important area for future research.

Methodological Critique and Heterogeneity

Several methodological issues should be considered when interpreting the findings. First, the included studies used related but not identical exposure definitions. Some studies focused on masked nocturnal hypertension, isolated nocturnal hypertension, nighttime masked uncontrolled hypertension, masked nighttime hypertension, nocturnal systolic blood pressure, or nocturnal dipping status. Although these phenotypes overlap clinically, they are not identical. For example, de la Sierra A et al., 2025, directly separated isolated daytime and isolated nighttime masked hypertension, while Ohkubo T et al., 2002, studied nocturnal blood pressure decline rather than masked nocturnal hypertension itself [25,30]. This distinction is important because directly classified masked nocturnal or isolated nocturnal hypertension studies answer the review question more closely, while broader ambulatory and nocturnal blood pressure studies provide supportive context.

Second, study populations differed substantially. CKD studies such as Fu X et al., 2022; Wang C et al., 2016; Li X et al., 2021; Drawz PE et al., 2016; Zhang J et al., 2021; and Do TM et al., 2025 showed strong associations with renal and cardiac outcomes, but these results may not be directly generalizable to lower-risk general populations [18,21,23,32,36,37]. Conversely, population-based studies such as Fan HQ et al., 2010; Ogedegbe G et al., 2013; Li Y et al., 2007; Booth JN 3rd et al., 2016; and Kim SH et al., 2022 provide broader external validity but sometimes have less detailed clinical phenotyping than specialist CKD or hypertension clinic cohorts. This heterogeneity supports narrative synthesis rather than quantitative pooling [20,27,29,31,34].

Third, many target-organ damage studies were cross-sectional. These studies are useful for detecting associations with left ventricular hypertrophy, arterial stiffness, albuminuria, or carotid intima-media thickness, but they cannot establish whether nocturnal hypertension preceded organ damage or whether organ damage contributed to nocturnal blood pressure dysregulation. Prospective evidence from Fan HQ et al., 2010; Wang C et al., 2016; de la Sierra A et al., 2025; and Booth JN 3rd et al., 2016, is therefore especially important because it supports temporality for cardiovascular and mortality outcomes [20,21,25,31].

Fourth, outcome definitions varied. Some studies reported hard outcomes such as cardiovascular events, stroke, cardiovascular mortality, all-cause mortality, renal events, or end-stage kidney disease. Others reported intermediate endpoints such as pulse wave velocity, carotid intima-media thickness, left ventricular mass index, or albuminuria. This mixture strengthens the biological narrative but makes the evidence unsuitable for a single pooled effect estimate.

Clinical Implications

The main clinical implication is that office blood pressure alone is insufficient to identify all adults at risk from hypertension-related cardiovascular and renal damage. The included studies repeatedly show that adults with normal or controlled office blood pressure may still have elevated nighttime blood pressure on ambulatory blood pressure monitoring and may carry a higher risk than truly normotensive individuals. This is especially relevant in CKD and type 2 diabetes, where masked nocturnal hypertension was common and associated with organ damage or adverse outcomes [17-19,21,23,26,32,37].

These findings support more targeted use of 24-hour ambulatory blood pressure monitoring in high-risk patients, even when office blood pressure appears controlled. Patients with CKD, diabetes, albuminuria, reduced estimated glomerular filtration rate, left ventricular hypertrophy, non-dipping patterns, or unexplained target-organ damage may benefit from ambulatory blood pressure monitoring to detect nighttime blood pressure elevation. However, the included studies do not provide enough interventional evidence to conclude that treating masked nocturnal hypertension improves outcomes. Therefore, ambulatory monitoring-based detection is supported, but the optimal treatment strategy, medication timing, and treatment targets require further trial evidence.

Limitations

This review should be interpreted in light of several limitations. Most included studies were observational, and many target-organ damage analyses were cross-sectional, which limits causal inference. Definitions of nighttime blood pressure and nocturnal hypertension differed between studies, including differences in nighttime windows, thresholds, and whether patients were classified by isolated nocturnal hypertension, masked nighttime hypertension, masked uncontrolled hypertension, or dipping status. Treatment status also varied, and few studies directly compared treated and untreated participants within the same analytic framework.

Some included studies were highly relevant but not perfectly aligned with the core masked nocturnal hypertension definition. Brguljan-Hitij J et al., 2014, supports the broader value of ambulatory blood pressure monitoring and masked hypertension for stroke risk, but the abstract focuses more on masked hypertension than isolated nocturnal hypertension [24]. Ohkubo T et al., 2002, supports the prognostic importance of nocturnal blood pressure decline but does not directly define masked nocturnal hypertension [30]. Zhang J et al., 2021, evaluated nocturnal systolic blood pressure levels in CKD rather than a strict masked nocturnal hypertension category [36]. These studies were retained as supportive evidence, but they should be interpreted separately from studies directly classifying masked nocturnal hypertension or isolated nocturnal hypertension.

Future Research

Future studies should use standardized definitions for masked nocturnal hypertension and isolated nocturnal hypertension, ideally based on accepted ambulatory blood pressure monitoring thresholds such as nighttime blood pressure ≥120/70 mmHg with normal or controlled office blood pressure. Prospective cohorts should report nocturnal hypertension subtypes separately from daytime and combined day-night hypertension, because de la Sierra A et al., 2025, suggest that the nocturnal subtype may carry a different mortality profile from daytime-only masked hypertension [25]. More studies are also needed in treated patients with controlled office blood pressure, especially in CKD and diabetes, to determine whether nocturnal blood pressure control changes the risk of left ventricular hypertrophy, renal progression, cardiovascular events, and mortality.

Interventional studies are particularly needed. The current evidence supports the detection of masked nocturnal hypertension, but it does not yet prove that treating this phenotype improves clinical outcomes. Trials comparing ambulatory blood pressure monitoring-guided management with usual office-based management would help determine whether nocturnal blood pressure treatment can reduce target-organ damage and hard cardiovascular endpoints.

## Conclusions

This systematic review shows that masked nocturnal hypertension and isolated nocturnal hypertension are clinically important blood pressure phenotypes that may remain undetected when assessment depends only on office blood pressure. Across the included studies, elevated nighttime blood pressure detected by 24-hour ambulatory blood pressure monitoring was linked with cardiovascular events, stroke, mortality, renal dysfunction, albuminuria, proteinuria, left ventricular hypertrophy, arterial stiffness, carotid intima-media thickness, and silent cerebrovascular lesions.

These findings suggest that normal or controlled office blood pressure does not always indicate low cardiovascular risk. Nighttime blood pressure may reveal a hidden burden of vascular, cardiac, renal, and cerebrovascular injury, particularly among patients with chronic kidney disease, diabetes mellitus, and treated hypertension. Overall, the evidence supports wider clinical consideration of ambulatory blood pressure monitoring in adults with apparently controlled office blood pressure, especially when cardiovascular or target-organ risk is suspected. Future prospective studies using standardized nocturnal blood pressure thresholds and consistent outcome definitions are needed to clarify prognosis and guide treatment decisions.
